# Адипоцитокины: современный взгляд на дефиницию, классификацию и роль в организме

**DOI:** 10.14341/probl12805

**Published:** 2021-12-06

**Authors:** Т. Н. Маркова, Н. К. Мищенко, Д. В. Петина

**Affiliations:** Городская клиническая больница №52 Департамента здравоохранения города Москвы; Московский государственный медико-стоматологический университет им. А.И. Евдокимова; Московский государственный медико-стоматологический университет им. А.И. Евдокимова; Городская клиническая больница №52 Департамента здравоохранения города Москвы

**Keywords:** жировая ткань, адипоцитокины, инсулинорезистентность, воспаление

## Abstract

Жировая ткань является эндокринным органом, синтезирующим большое количество биологически активных веществ — адипоцитокинов, влияющих на инсулинорезистентность (ИР), метаболизм глюкозы и липидов, процессы ангиогенеза и воспаления. Данные многочисленных исследований показывают тесную связь дисбаланса адипоцитокинов, формирующегося при избыточном отложении жировой ткани, с развитием сахарного диабета 2 типа и сердечно-сосудистых заболеваний. В статье представлен обзор современной литературы, обобщающий данные о воздействии адипоцитокинов на печень, скелетные мышцы, жировую ткань, эндотелиальные клетки и процессы воспаления, а также представлена попытка дефиниции термина «адипоцитокины» и классификации адипоцитокинов с позиции их влияния на метаболические сдвиги и провоспалительный статус. Представители адипоцитокинов (адипонектин, оментин, лептин, резистин, фактор некроза опухоли-α и интерлейкин-6) разделены на две группы: адипоцитокины, снижающие ИР, и адипоцитокины, повышающие ИР.

## Abstract (en)

Adipose tissue is an endocrine organ which produces a large number of secretory bioactive substances also known as adipocytokines affecting directly insulin resistance (IR), glucose and lipid metabolism, angiogenesis and inflammation. The studies show a close connection between the imbalance of adipocytokines formed as a result of excessive deposit of adipose tissue in the course of the development of type 2 diabetes mellitus and cardiovascular diseases. In the present review, we summarize current data on the effect of the adipocytokines on the liver, skeletal muscles, adipose tissue, endothelial cells and inflammatory processes, as well as attempt to define the term «adipocytokines» and classify adipocytokines according to their influence on metabolic processes and pro-inflammatory status. Some of adipocytokines (adiponectin, omentin, leptin, resistin, tumor necrosis factor-α and interleukin-6) are divided into two groups: adipocytokines reducing IR, and adipocytokines increasing IR.

## МЕТОДОЛОГИЯ ПОИСКА ИСТОЧНИКОВ

В процессе написания статьи использовались следующие базы данных: www.elibrary.ru, www.ncbi.nlm.nih.gov/pubmed, www.clinicalTrials.gov, поисковая система Google. Поиск проводился по ключевым словам: жировая ткань, адипоцитокины, инсулинорезистентность, воспаление.

## ВВЕДЕНИЕ

В течение последних двух десятилетий все большее внимание уделяется изучению жировой ткани как эндокринного органа, способного вырабатывать адипоцитокины — биологически активные вещества, синтезируемые жировой тканью и обладающие многочисленными метаболическими эффектами [1]. В данном определении есть несколько неточностей. Во-первых, практически все адипоцитокины вырабатываются не только жировой тканью, но и другими тканями, тогда, вероятно, термин «адипоцитокин» не в полной мере отражает действительность, во-вторых, необходимо провести некоторую дифференцировку между адипокинами и адипоцитокинами. Термин «адипоцитокин» признается не всеми учеными, поскольку цитокины — вещества, участвующие в иммуновоспалительных реакциях. Однако не все адипоцитокины обладают иммуномодулирующим действием [2]. На сегодня в научной литературе наряду с термином «адипоцитокин» выделяют понятие «адипокин». В настоящее время не разработаны четкие дефиниции данных терминов. Так, H. Cao (2014) описывает адипоцитокины как вещества, синтезированные в жировой ткани вследствие взаимодействия между адипоцитами и иммунными клетками. Схема взаимодействия адипоцита и иммунных клеток, расположенных в жировой ткани, представлена на рисунке 1 [2].

**Figure:**
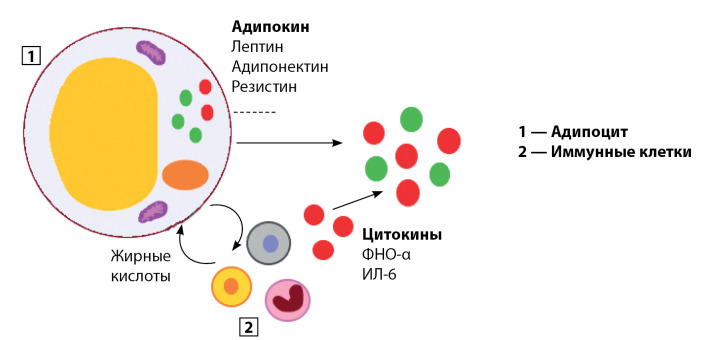
Рисунок. Схема взаимодействия адипоцита и иммунных клеток в жировой ткани [адаптировано 2].

В то же время Booth A. et al. (2015) не выделяют различий между терминами «адипоцитокин» и «адипокин» [3]. Следовательно, биологически активные вещества, продуцируемые жировой тканью, могут быть представлены как адипоцитокинами, так и адипокинами.

Современное представление о классификации адипоцитокинов основывается на их воздействии на клетки-мишени. Так, Ouchi N. et al. (2011) выделяют две группы адипоцитокинов: провоспалительные и противовоспалительные [4]. Согласно классификации Oh K.J. et al. (2016), адипоцитокины можно разделить на воспалительные, противовоспалительные и другие субстанции [5]. По данным литературы, лишь единичные статьи указывают на возможность классификации адипоцитокинов согласно месту их синтеза. Так, Чубриева С.Ю. и соавт. (2008) разделяют данные вещества на специфичные и неспецифичные для жировой ткани адипоцитокины [6]. В таблице 1 суммированы несколько классификаций адипоцитокинов.

**Table:** Таблица 1. Классификация адипоцитокинов

Автор	Ouchi N. et al. [4]	Oh K.J. et al. [5]	Чубриева С.Ю. [6]
Группы	1. Провоспалительные:-лептин-резистин-ретинол-связывающий белок-липокалин-2-ангиопоэтин-подобный белок-2-фактор некроза опухоли альфа-интерлейкин-6-интерлейкин-18-хемокины (CCL2, CXCL5)-висфатин	1. Провоспалительные:-лептин-резистин-фактор некроза опухоли альфа-интерлейкин-6-ретинол-связывающий белок	1. Специфичные для жировой ткани:-адипонектин-лептин
2. Противовоспалительные:-адипонектин-секретируемый человеческий протеин-5, родственный белкам Frizzled	2. Противовоспалительные:-адипонектин-фактор роста фибробластов-21-секретируемый человеческий протеин-5, родственный белкам Frizzled	2. Неспецифичные для жировой ткани:-ингибитор-1 активатора плазминогена-фактор некроза опухоли альфа
	3. Другие:-иризин-фетуин-А	

В настоящем обзоре представлена классификация адипоцитокинов с позиции их влияния на инсулинорезистентность (ИР). Семь представителей адипоцитокинов разделены на две группы: адипоцитокины, снижающие ИР, и адипоцитокины, повышающие ИР. В 2005 г. Международная ассоциация диабета (IDF) выделила понятие ИР в качестве основного звена патогенеза метаболического синдрома (МС), включающего в себя абдоминальное ожирение, дислипидемию, нарушения углеводного обмена и артериальную гипертензию [7]. МС является фактором риска развития сахарного диабета 2 типа (СД2) и сердечно-сосудистых заболеваний (ССЗ). Изучение биологически активных веществ, снижающих или повышающих ИР, позволит разработать стратегии лечения данных заболеваний.

## АДИПОЦИТОКИНЫ, СНИЖАЮЩИЕ ИНСУЛИНОРЕЗИСТЕНТНОСТЬ

К группе адипоцитокинов, снижающих ИР, отнесены адипонектин, оментин и лептин.

## АДИПОНЕКТИН

Адипонектин представляет собой белок, состоящий из 244 аминокислот и имеющий коллагеноподобный участок. Данный адипоцитокин циркулирует в плазме крови в виде различных изоформ: низкомолекулярного тримера, среднемолекулярного гексамера и высокомолекулярного олигомера, среди которых биологически активной формой гормона является высокомолекулярный олигомер. В настоящее время получены данные, что адипонектин синтезируется не только адипоцитами жировой ткани, но и другими клетками, включая остеобласты, клетки паренхимы печени, миоциты, эпителиальные клетки и плацентарную ткань [8]. Адипонектин взаимодействует с двумя типа рецепторов: AdipoR1 и AdipoR2. AdipoR1 расположен во многих тканях организма, преимущественно в скелетных мышцах. AdipoR2 представлен главным образом в клетках печени [8]. С помощью данных рецепторов адипонектин оказывает многочисленные метаболические эффекты.

Так, в исследованиях показано, что под действием адипонектина в скелетных мышцах происходит усиление поглощения глюкозы, а также активируется процесс окисления жирных кислот. При изучении эффектов адипонектина на гепатоциты выявлено подавление процессов глюконеогенеза и гликогенолиза. Кроме того, адипонектин способен повышать активность карнитин-пальмитоилтрансферазы I и усиливать окисление жирных кислот в митохондриях клеток печени, одновременно уменьшая активность ключевых ферментов, участвующих в синтезе жирных кислот. Снижение уровня жирных кислот в гепатоцитах приводит к уменьшению ИР, так как триглицериды являются антагонистами инсулина [9].

Высокая экспрессия адипонектина отмечается не только в скелетных мышцах и печени, но и в жировой ткани. Благодаря своей аутокринной активности адипонектин способствует дифференцировке клеток адипоцитов, стимулирует адипогенез, повышает содержание липидов в адипоцитах, а также усиливает инсулин-направленный транспорт глюкозы [10]. Особый интерес представляет изучение влияния адипонектина на метаболические процессы у мышей, лишенных лептина, с гиперфагией (мыши ob/ob). В данном исследовании повышенный уровень адипонектина вызывал перераспределение триглицеридов из печени и мышечной ткани в жировую ткань, что улучшало чувствительность тканей к инсулину и приводило к нормализации уровня глюкозы крови и инсулина. Одновременно наблюдалось заметное улучшение метаболизма глюкозы в жировой ткани, сопровождающееся уменьшением количества макрофагов и снижением экспрессии фактора некроза опухоли альфа (TNFα). Кроме того, избыточная экспрессия адипонектина у мышей ob/ob приводила к увеличению количества подкожножировой клетчатки и усилению ее васкуляризации. В целом можно сделать вывод, что повышенное количество адипонектина приводит к массивному увеличению количества подкожножировой клетчатки и предотвращает развитие ИР, обусловленное избыточным потреблением пищи [11].

Особый интерес представляет ассоциация уровня адипонектина и количества висцеральной жировой ткани. Так, известно, что концентрация данного адипоцитокина в сыворотке крови отрицательно коррелирует с количеством висцеральной жировой ткани [12]. По данным Reneau J., cекреция адипонектина висцеральной жировой ткани уменьшается с увеличением степени ожирения, в то время как секреция изучаемого адипокина подкожно жировой клетчаткой остается неизменной, особенно у женщин. Это наблюдение может объяснить более низкие уровни циркулирующего адипонектина у лиц с центральным типом ожирения [13].

Важное значение имеет влияние адипоцитокина на поджелудочную железу. Так, результаты исследований показали, что введение адипонектина тучным мышам подавляет высвобождение инсулина при базальных концентрациях глюкозы и усиливает секрецию инсулина при ее повышенной концентрации. Также в опытах на мышах продемонстрирована способность адипонектина предотвращать апоптоз β-клеток поджелудочной железы, вызванный избыточным воздействием липидов и цитокинов [14].

Адипонектин оказывает влияние на сосудистый гомеостаз, воздействуя на важные сигнальные пути в эндотелиальных клетках и модулируя воспалительные реакции в субэндотелиальном пространстве. Влияние данного адипокина на сердечно-сосудистую систему частично опосредовано увеличением активности 5’-аденозинмонофосфат-активируемой протеинкиназы, что приводит к повышению концентрации оксида азота и предотвращает апоптоз эндотелиальных клеток. Также адипонектин препятствует активации эндотелиальных клеток после стимуляции провоспалительными веществами, такими, как TNFα и ядерный фактор каппа-в [15].

Таким образом адипонектин можно отнести к адипоцитокинам, снижающим ИР и обладающим противовоспалительным и антиатерогенным действиями. Благодаря описанным выше метаболическим эффектам, содержание данного адипокина исследуется у пациентов с СД2 [9, 16], ССЗ [9] и ожирением [16].

## ОМЕНТИН

Оментин (интелектин-1) — белок, состоящий из 313 аминокислотных остатков. Данное вещество вырабатывается преимущественно стромальными сосудистыми клетками висцеральной жировой ткани [17]. Также он может синтезироваться в эндотелиальных клетках, эпителиальных клетках кишечника [17], эпикардиальной жировой ткани, легких, яичниках и плаценте [18]. В настоящее время специфические рецепторы оментина не идентифицированы [19].

В исследованиях in vitro показано, что оментин повышает чувствительность адипоцитов человека к инсулину. Так, рекомбинантный оментин индуцировал фосфорилирование протеинкиназы В и усиливал стимулированное инсулином поглощение глюкозы как в подкожножировой клетчатке, так и в висцеральной жировой ткани [17]. Влияние оментина на скелетные мышцы до конца не изучено. Согласно данным литературы, в настоящее время не проведены крупные исследования, направленные на определение его функциональной роли в скелетных мышцах с использованием моделей грызунов или тканей человека [20].

Также большой интерес представляет влияние оментина на эндотелиальные клетки. В исследованиях определено снижение активности TNFα под влиянием оментина, что в дальнейшем приводит к уменьшению синтеза молекул адгезии [21][22]. Кроме того, в опытах на крысах, страдающих СД2, выявлено улучшение эндотелиальной дисфункции под воздействием оментина [23]. Таким образом, оментин оказывает противовоспалительное действие на сосудистую стенку [22].

В исследованиях in vivo уровень оментина в плазме крови обратно коррелировал с индексом массы тела и показателями ИР, положительно — с содержанием адипонектина и холестерина липопротеинов высокой плотности. Таким образом, снижение уровня оментина связано с увеличением риска развития ожирения и ИР [24]. Также, по данным исследований, выявлена взаимосвязь между концентрацией оментина и нарушениями углеводного обмена и ССЗ. Так, достоверное снижение уровня оментина зафиксировано у пациентов с СД2 [25].

В целом оментин можно классифицировать как адипоцитокин, снижающий ИР. Однако необходимы дальнейшие исследования, уточняющие физиологическую роль оментина в углеводном обмене.

## ЛЕПТИН

Лептин — белок, состоящий из 146 аминокислот [26]. Данный адипокин синтезируется преимущественно жировой тканью и в небольшом количестве слизистой дна желудка [27]. Cтруктура лептина сходна по своему строению с провоспалительными цитокинами, такими как интерлейкин 6 (ИЛ-6) и гранулоцитарный колониестимулирующий фактор [26]. Лептин опосредует свои эффекты, связываясь со специфическими рецепторами (ObR), экспрессируемыми в головном мозге, а также в периферических тканях (нервной ткани, печени, поджелудочной железе, сердце и кишечнике) [28].

Главным органом-мишенью лептина служит дугообразное ядро гипоталамуса. Данное ядро играет важную роль в регулировании аппетита и энергетического гомеостаза. Нейроны дугообразного ядра содержат орексигенные (агути-подобный белок/нейропептид Y) и анорексигенные пептиды (проопиомеланокортин). Связывание лептина с нейронами дугообразного ядра приводит к высвобождению проопиомеланокортина и ингибированию выделения агути-подобного белка/нейропептида Y, что вызывает снижение аппетита [28].

Лептин также воздействует на печень, скелетные мышцы и жировую ткань. Так, по результатам исследований обнаружено, что описанный белок ограничивает накопление триглицеридов в печени и скелетных мышцах. Кроме того, лептин модулирует функцию β-клеток поджелудочной железы. По мере увеличения массы тела повышенная выработка данного вещества приводит к предотвращению накоплению липидов в β-клетках. Также в исследованиях на животных показано, что лептин ингибирует секрецию инсулина поджелудочной железой [29]. Влияние лептина на сосудистую стенку до конца не изучено. В литературе имеются данные, сообщающие как о проангиогенном, так и об антиангиогенном действии описанного вещества [30]. Исследования, оценивающие роль лептина в иммунных реакциях, показали провоспалительное действие данного адипоцитокина [31]. Так, показано, что лечение лептином увеличивало выработку моноцитами цитокинов 1 типа, включая ИЛ-1, ИЛ-6 и TNFα, а также резистин [32].

Известным является факт, что у лиц с ожирением часто наблюдается лептинорезистентность. В ее основе лежат изменения транспорта лептина через гематоэнцефалический барьер, нарушение передачи сигнала от данного белка к рецепторам и, как следствие, снижение синтеза нейротрансмиттеров и нейромедиаторов в ответ на действие лептина. Ослабление чувствительности к описанному адипоцитокину в головном мозге приводит к избыточному накоплению триглицеридов в жировой ткани, а также в мышцах, печени и поджелудочной железе, что в конечном итоге нарушает секрецию инсулина [29].

Лептин подавляет потребление пищи и способствует расходованию энергии. Независимо от этих эффектов, лептин улучшает чувствительность периферических тканей (печени и скелетных мышц) к инсулину и модулирует функцию β-клеток поджелудочной железы. В большинстве случаев у пациентов с ожирением, несмотря на высокий уровень циркулирующего в кровеносном русле лептина, не происходит снижения веса, что отражает наличие лептинорезистентности [29].

В диагностическом плане лептин служит критерием диагностики редких форм ожирения. Так, согласно российским рекомендациям по лечению ожирения и коморбидных заболеваний, определение уровня данного адипоцитокина проводится при подозрении на моногенные формы ожирения [33].

## АДИПОЦИТОКИНЫ, ПОВЫШАЮЩИЕ ИНСУЛИНОРЕЗИСТЕНТНОСТЬ

В группу адипоцитокинов, повышающих ИР, отнесены резистин, ретинол-связывающий протеин 4 (RBP4) и маркеры воспаления (TNFα, ИЛ-6).

## РЕЗИСТИН

Резистин — низкомолекулярный белок, принадлежащий семейству резистиноподобных молекул. Первоначально данный адипоцитокин был выделен из жировой ткани, однако позже обнаружено, что основными источниками синтеза резистина являются макрофаги [34]. Резистин выявлен и в других органах и тканях: в костном мозге, легких, плаценте и β-клетках поджелудочной железы [35]. В настоящее время специфические рецепторы резистина не обнаружены [34].

В исследованиях на животных показано, что введение резистина мышам, страдающим ожирением, приводило к развитию нарушенной толерантности к глюкозе. В то же время уменьшение концентрации описываемого адипоцитокина снижало уровень глюкозы крови у мышей с ожирением и улучшало толерантность к глюкозе у здоровых животных. Данный белок снижал поглощение глюкозы скелетными мышцами независимо от активируемых инсулином сигнальных путей [36]. Кроме того, в опытах на крысах под влиянием резистина выявлено усиление активности апоптоза β-клеток поджелудочной железы при наличии инсулиномы [37]. Также показано, что описанный адипоцитокин в гепатоцитах крыс приводил к замедлению процессов гликогенеза и усилению гликогенолиза. Таким образом, содержание гликогена в печени уменьшалось [38].

В то же время влияние резистина на развитие гиперинсулинемии и ИР у людей до конца не изучено. В настоящее время в литературе имеются данные, свидетельствующие как о положительной корреляции между уровнями резистина и ожирением или ИР [39], так и об отсутствии изменений концентрации данного белка при ожирении, ИР и СД2 [40]. Однако большинство ученых описывают резистин как потенциальный фактор развития ИР и СД2. Так, показано, что специфические нуклеотидные полиморфизмы в гене RETN, кодирующем синтез резистина, вызывают ожирение, ИР и СД2 [41]. В исследованиях выявлен высокий уровень резистина в сыворотке крови у пациентов с ожирением и зафиксирована прямая корреляционная связь между уровнем данного адипоцитокина и массой жировой ткани [41].

Кроме влияния резистина на ИР, выявлены и другие эффекты описанного белка. Так, резистин принимает участие в процессах воспаления и дисфункции эндотелия [41][42]. Резистин обладает способностью активировать эндотелий, стимулируя высвобождение эндотелина-1, а также нарушает регуляцию молекул адгезии и хемокинов в сосудистых клетках [41]. Кроме того, отмечена способность резистина проявлять сильные провоспалительные свойства за счет активации секреции TNFα, ИЛ-6, ИЛ-12, моноцитарного хемотаксического протеина-1 через NF-kB-опосредованный путь [42].

В настоящее время резистин рассматривается как белок, усиливающий ИР. Данный адипоцитокин способен усугублять развитие клинических состояний, таких как ожирение, СД2 и ССЗ.

## РЕТИНОЛ-СВЯЗЫВАЮЩИЙ ПРОТЕИН 4 (RBP4)

Ретинол-связывающий протеин 4 (RBP4) — белок, относящийся к семейству транспортных белков липокалинов и переносящий в кровеносном русле ретинол, известный как витамин А. Экспрессия данного вещества наиболее высока в печени, где содержится основное количество витамина А в организме. Однако RBP4 синтезируется в жировой ткани и в меньшем количестве в почках, пигментном эпителии сетчатки, яичках, легких и сосудистом сплетении желудочков головного мозга [43]. В настоящее время идентифицированы два рецептора RBP4 (STRA6 и RBPR2), которые опосредуют поглощение и высвобождение ретинола через клеточную мембрану [43][44].

Основная биологическая роль RBP4 заключается в транспортировке ретинола из печени в органы-мишени [43]. Однако в литературе имеются данные, подтверждающие взаимосвязь между повышенным уровнем описанного белка и развитием ИР и СД2. Так, полиморфизм гена RBP4, приводящий к увеличению экспрессии RBP4 в жировой ткани [45], повышает риск развития СД2 у людей [46].

В настоящее время наиболее детально изучено влияние данного адипоцитокина на метаболизм глюкозы и липидов у животных. Так, искусственно индуцированное повышение уровня RBP4 вызывает резистентность к инсулину [47], а снижение концентрации данного вещества у тучных мышей улучшает чувствительность к инсулину [48]. Повышение уровня RBP4 увеличивает экспрессию основного фермента глюконеогенеза — фосфоенолпируваткарбоксикиназы — в печени и нарушает действие инсулина в скелетных мышцах [47]. Гиперэкспрессия RBP4 в жировой ткани мышей приводит к активации липолиза с последующим высвобождением свободных жирных кислот в кровеносное русло, а также к усилению процессов воспаления в жировой ткани [49].

Исследования влияния RBP4 на маркеры ИР у людей ограничены. Так, в работе Kilicarslan M. et al. показано, что содержание RBP4 в печени, висцеральной жировой ткани и подкожной жировой клетчатке повышено у пациентов с морбидным ожирением. Концентрация RBP4 в кровеносном русле обратно коррелирует с инсулинозависимым подавлением липолиза и эндогенной продукцией глюкозы и утилизацией глюкозы периферическими тканями вследствие нарушения действия инсулина в скелетных мышцах. Кроме того, имеется связь между уровнями мРНК RBP4 и показателями мРНК маркеров воспаления в жировой ткани. Данный адипоцитокин оказывает свое действие прямо и косвенно через активацию макрофагов с помощью повышения уровня TNFα [50]. Также по результатам исследования Васюка Ю.А. и соавт. выявлено негативное влияние RBP4 на кардиометаболические риски у пациентов с артериальной гипертензией и ожирением [51].

Таким образом, RBP4 — не только белок, участвующий в транспорте ретинола, но и вещество, регулирующее обмен глюкозы и липидов, а также участник иммунных реакций.

## МАРКЕРЫ ВОСПАЛЕНИЯ

Согласно данным литературы, при наличии ожирения отмечается значительная инфильтрация жировой ткани макрофагами, способными секретировать провоспалительные цитокины (TNFα, ИЛ-6 и другие). Кроме того, под действием TNFα преадипоциты начинают выработку провоспалительных цитокинов, способствуя дальнейшей активации макрофагов. Таким образом, формируется порочный круг, характеризующийся снижением чувствительности адипоцитов к инсулину [52].

## ФАКТОР НЕКРОЗА ОПУХОЛЕЙ АЛЬФА (TNFα)

Фактор некроза опухолей альфа (TNFα) — провоспалительный цитокин, секретируемый преимущественно макрофагами [53]. По данным литературы известно, что TNFα снижает экспрессию инсулинозависимого транспортера глюкозы — ГЛЮТ-4, который локализуется в адипоцитах, скелетных мышцах и миокарде [54]. Кроме того, данный цитокин, индуцируя фосфорилирование серина в субстрате инсулинового рецептора-1, ингибирует действие инсулина [55]. В адипоцитах TNFα стимулирует липолиз, увеличивая уровень свободных жирных кислот [55]. Кроме того, TNFα обладает прямым провоспалительным эффектом в отношении сосудистых клеток, вызывая развитие дисфункции эндотелия [56].

Метаанализ исследований показал, что уровень TNFα в сыворотке крови значительно повышен как у пациентов с СД1, так и у лиц с СД2, что отражает высокий провоспалительный статус при СД в целом и связь ИР с воспалением при СД2 [57][58]. Применение ингибиторов TNFα у лиц с метаболическим синдромом привело к снижению уровня гликемии натощак и повышению концентрации адипонектина [59]. Таким образом, TNFα играет важную роль в развитии ИР.

## ИНТЕРЛЕЙКИН-6 (ИЛ-6)

Интерлейкин-6 (ИЛ-6) — цитокин, который синтезируется главным образом макрофагами [52]. В скелетных мышцах во время физических упражнений ИЛ-6 увеличивает поглощение глюкозы, что приводит к гипертрофии мышц, миогенезу и окислению жирных кислот, а также оказывает противовоспалительное действие. В то время как в жировой ткани и печени данный белок усиливает ИР. Так, ИЛ-6 снижает индуцированное инсулином фосфорилирование субстрата инсулинового рецептора-1, вследствие чего тормозится захват глюкозы адипоцитами и усиливается липолиз [55]. Также показано, что повышение концентрации ИЛ-6 наблюдается у пациентов с СД2. В целом, оценивая суммарное воздействие данного цитокина на биохимические процессы, ИЛ-6 можно отнести к веществам, усиливающим ИР.

## ЗАКЛЮЧЕНИЕ

Адипоцитокины — это биологически активные вещества, вырабатываемые преимущественно жировой тканью и другими тканями организма, влияющие на метаболические процессы, в первую очередь на жировой и углеводный обмены. На наш взгляд, все описанные в статье биологически активные вещества с учетом их влияния на воспалительный статус являются адипоцитокинами, поэтому нецелесообразно выделять отдельный термин «адипокины». С учетом их основных механизмов действия имеет смысл классифицировать адипоцитокины в группы в зависимости от влияния на метаболические эффекты и воспалительный процесс. Попытка суммировать эффекты адипоцитокинов представлена в таблице 2.

**Table:** Таблица 2. Классификация адипоцитокинов в зависимости от основных эффектов

Адипоцитокины	Метаболические эффекты	Влияние на воспалительный процесс
Снижающие ИР
Адипонектин	-Подавление глюконеогенеза, гликогенолиза в печени-Стимуляция окисления жирных кислот в печени и скелетных мышцах-Стимуляция поглощения глюкозы в печени и скелетных мышцах-Стимуляция секреции инсулина	Снижает
Оментин	-Усиление поглощения глюкозы в адипоцитах	Снижает
Лептин	-Стимуляция окисления жирных кислот в печени, поджелудочной железе и скелетных мышцах-Модуляция функции β-клеток поджелудочной железы	Повышает
Повышающие ИР
Резистин	-Снижение захвата глюкозы скелетными мышцами, усиление гликогенолиза, апоптоз β-клеток в опытах на животных	Повышает
RBP4	-Снижение захвата глюкозы скелетными мышцами, усиление глюконеогенеза, стимуляция липолиза в опытах на животных	Повышает
TNFα	-Модуляция действия инсулина в печени и скелетных мышцах-Снижение захвата глюкозы скелетными мышцами, жировой тканью-Стимуляция липолиза в жировой ткани	Повышает
ИЛ-6	-Модуляция действия инсулина в печени и скелетных мышцах-Снижение захвата глюкозы жировой тканью-Стимуляция липолиза в жировой ткани	Повышает

Дальнейшие исследования, оценивающие физиологическую роль адипоцитокинов, помогут расширить диагностические критерии оценки уровня данных веществ в кровеносном русле и, возможно, позволят создать терапевтические средства для лечения патологических состояний, таких как ожирение, СД2 и ССЗ.

## ДОПОЛНИТЕЛЬНАЯ ИНФОРМАЦИЯ

Источник финансирования. Работа выполнена по инициативе авторов без привлечения финансирования.

Конфликт интересов. Авторы декларируют отсутствие явных и потенциальных конфликтов интересов, связанных с содержанием настоящей статьи.

Участие авторов. Маркова Т.Н. — концепция и дизайн статьи, проверка статьи, внесение коррективов, утверждение рукописи; Мищенко Н.К. — дизайн статьи, сбор и обработка материалов, написание текста; Петина Д.В. — сбор и обработка материалов, написание текста. Все авторы одобрили финальную версию статьи перед публикацией, выразили согласие нести ответственность за все аспекты работы, подразумевающую надлежащее изучение и решение вопросов, связанных с точностью или добросовестностью любой части работы.
